# The Effect of Freeze/Thaw Cycles on Reproducibility of Metabolic Profiling of Marine Microalgal Extracts Using Direct Infusion High-Resolution Mass Spectrometry (HR-MS)

**DOI:** 10.3390/molecules191016373

**Published:** 2014-10-13

**Authors:** Hans Chr. Eilertsen, Siv Huseby, Maria Degerlund, Gunilla K. Eriksen, Richard A. Ingebrigtsen, Espen Hansen

**Affiliations:** 1 Department of Arctic and Marine Biology, Faculty of Biosciences, Fisheries and Economics, UiT The Arctic University of Norway, Tromsø N-9037, Norway; E-Mails: hans.c.eilertsen@uit.no (H.C.E.); maria.degerlund@uit.no (M.D.); gunilla.eriksen@uit.no (G.K.E.); richard.a.ingebrigtsen@uit.no (R.A.I.); 2 MabCent-SFI, UiT The Arctic University of Norway, Tromsø N-9037, Norway; E-Mail: siv.huseby@uit.no; 3 Marbio, UiT The Arctic University of Norway, Tromsø N-9037, Norway

**Keywords:** extracts, metabolic profiling, mass spectrometry, freeze/thaw cycles, stability

## Abstract

During normal sample preparation, storage in freezers and subsequent freeze/thaw cycles are commonly introduced. The effect of freeze/thaw cycles on the metabolic profiling of microalgal extracts using HR-MS was investigated. Methanolic extracts of monocultures of Arctic marine diatoms were analyzed immediately after extraction, after seven days of storage at −78 °C (one freeze/thaw cycle), and after additional seven days at −20 °C (two freeze/thaw cycles). Repeated direct infusion high-resolution mass spectrometry analysis of microalgae extracts of the same sample showed that reproducibility was *ca.* 90% when a fresh (unfrozen) sample was analyzed. The overall reproducibility decreased further by *ca.* 10% after the first freeze/thaw-cycle, and after one more freeze/thaw cycle the reproducibility decreased further by *ca.* 7%. The decrease in reproducibility after freeze-thaw cycles could be attributed to sample degradation and not to instrument variability.

## 1. Introduction

Over the last two decades the use of mass spectrometry (MS) for quantitative and qualitative analysis of biological molecules has been increasingly applied in different fields of biological research, not only in medical biology and pharmacology but also in ecology and taxonomy [1,2].

Thus, MS has become an important analytical tool in many biological labs. This is partly due to the improved versatility, reliability and availability of modern MS-instruments. Furthermore, improvements in mass filters and ion optics have made it possible to acquire accurate mass data at high resolution with relatively affordable bench top instruments [3]. This has also greatly facilitated the identification of unknown compounds [4]. However, as more sophisticated and sensitive MS instruments have become available, it is important to communicate the limitations related to such methods and their respective causes.

Metabolic fingerprinting is the high-throughput global analysis of metabolites to provide sample classification [5]. It has also been used to classify yeast and bacteria [6], and metabolomics approaches will become increasingly more important in the future, especially when “genetics meets metabolomics” [7]. When performing metabolic fingerprinting based on HR-MS data two different strategies are possible, *i.e.*, either a direct infusion of the sample into the MS or a chromatographic separation of the compounds prior to analysis in the MS [8]. Both gas and liquid chromatography (GC and LC) can be used for metabolic profiling [9]. Chromatographic separation offers some advantages over direct infusion, such as reduced risk of ion suppression and facilitated metabolite annotation based on isotopic distribution and recognition of adducts. However, direct infusion MS is preferred in many metabolomic investigations because it offers high sample throughput, which is crucial when analyzing large numbers of samples. Direct infusion in metabolomics also benefits from HR-MS since compounds with different elemental compositions but identical nominal masses will be separated [8].

In on-going research projects in our laboratory, we frequently apply non-targeted metabolomics (*i.e.*, metabolic fingerprinting) on extracts from diatom samples [10,11]. As we analyze large numbers of species, we use direct infusion HR-MS. Due to restrictions in capacity, common to most other laboratories, we store samples in both bio-freezers (−78 °C) and in ordinary freezers (−20 °C). There are several studies of the reproducibility and robustness of LC-MS and GC-MS methods used in metabolomics [12,13], but information on the effect of freeze/thaw cycles on biological extracts is scarce. In order to test the reproducibility of our direct infusion HR-MS method prior to and after freezing, we performed repeated analyses on extracts from the same diatom sample.

## 2. Results and Discussion

When analyzing the extracts by direct infusion HR-MS, we observed approximately the same number of *m/z* signals (1896–1901) in the different samples. After removal of noise (see Experimental Section) we had between 1409 and 1420 *m/z* values from each sample left for processing. We compared the number of common *m/z* signals between the samples. If a compound in a sample was chemically altered during the experiment there would be a mass change and it would appear as a different *m/z* signal, thus decreasing the number of common signals between the two analyses. We did not observe any decrease in the total number of *m/z* signals in each analysis even though the number of common *m/z* signals did decrease significantly throughout the experiment (see below). The relative standard deviations (RSDs) between the repeated analyses of each sample were minor (2.8%–6.1%), and on average they increased slightly during the experiment (Table 1).

**Table 1:** Number of identical *m/z* signals between repeated analyses of each sample. A, B and C refers to the three cultivation replicates, RSD at *p* = 0.05 (in parentheses), n = number of comparisons.

Analysis	Storage	Number of Freeze/Thaw Cycles	Number of Identical *m/z* Signals	n
First	Fresh sample	0	89.6% (3.6)	30
Second	One week at −78 °C	1	A 80.7% (2.8)	10
			B 79.2% (4.2)	10
			C 80.3% (3.1)	10
			Average 80.1%	
Third	One additional week at −20 °C	2	A 67.3% (3.4)	10
			B 76.7% (6.1)	10
			C 74.4% (3.9)	10
			Average 72.8%	

The first analyses performed on fresh samples (n = 30) showed reproducibility in the detection of common *m/z* signals of 89.6%, *i.e.*, there was a 10.4% difference between the samples (Table 1). When filters where frozen and stored at −78 °C for one week and subsequently thawed, extracted and analyzed by MS, we observed an additional decrease in reproducibility of 9.5% compared to the first analysis (Table 1). The third analysis (extracts from second analysis stored at −20 °C) showed that reproducibility decreased further to a mean of 72.8%.

Modern HR-MS instruments commonly have a mass accuracy between 1 and 5 ppm, but the accuracy of the MS in direct infusion of complex mixtures is reduced compared to introducing chromatographically separated compounds into the MS. This is due to the complexity of the ion populations that will be simultaneously accelerated into the MS using direct infusion, and that centroiding of the continuous data produced by the MS will be affected by *m/z* signals that are close to each other. The internal calibration of the instrument during data acquisition was done using leucine-enkapheline, with a protonated mass of 556.2771 Da, as lock mass. Thus, the mass accuracy will be highest for *m/z* signals close to this mass. We believe that the 89.6% reproducibility of the fresh sample is a result of introducing the complex samples to the MS via direct infusion. The binning of the continuous data from the MS into discrete *m/z* signals might be affected by small changes in intensity and mass of a complex sample, and this will reduce the reproducibility compared to a LC-MS analysis where the complexity of the samples reaching the ion source will be much less. Our results indicate that a maximum of 90% reproducibility can be achieved at 150 ppm accuracy when analyzing fresh extracts using direct infusion HR-MS. 

When performing a high throughput type of analysis it is often necessary to store samples frozen, and it is known that storage may affect the chemical integrity of biological samples [14]. This may be related to the storage temperature, the nature of the samples in question as well as the storage solvent. A thorough test of the effect of freeze/thaw cycles on 320 organic compounds showed that the specific amounts decreased 3%–4% after each freeze/thaw cycle and that this increased in a linear manner when the freeze thaw cycles were repeated [15]. One implication might be that it is the number of freeze/thaw cycles that causes compound degradation or precipitation, and the duration of the storage in a frozen state is less important. Our samples were stored frozen prior to the second analysis, as is common procedure is in our lab due to extraction and MS capacity. If we conclude that the instrument and methodological basic error is *ca.* 10%, our results demonstrate a 7%–10% decrease in reproducibility for each of the first two freeze/thaw cycles. The decrease in reproducibility was largest during the first freeze/thaw cycle when filters were stored at −78 °C (10% decrease) while keeping the extracts at −20 °C caused a further decrease of *ca.* 7%. This observed decrease in reproducibility associated with storage is probably partly due to freeze-thaw cycles (as discussed above), but may also be a result of degradation of compounds during the storage itself. Proteins may be denaturated and some lipids may be easily oxidized if a stable (an inert) atmosphere is not applied [16].

Our intention with splitting the original cultures and keeping them in three separate bottles was to test the influence of minor differences in cultivation conditions, e.g., a “bottle effect” [17] or metabolic effects of variable growth phases [18]. The analyses performed on each of these bottles (A, B, C) yielded approximately the same number of hits (80.7%, 79.2% and 80.3%) and SDs were minor (Table 1). It was therefore no reason to conclude that variations in culture conditions or sample handling during extraction had much influence on the outcome of our analyses after the first freeze/thaw cycle. Thus, the observed increase in variation between the samples in the third analysis (67.3%, 76.7% and 74.4%) can not be attributed to the cultivation conditions but to the second freeze/thaw cycle and sample storage.

## 3. Experimental Section

### 3.1. Sample Preparation

A monoculture of the northern diatom *Porosira glacialis* (Grunow) Jørgensen was isolated (manual retrieval) from a culture originating from Tromsøysund outside the city of Tromsø in northern Norway. *P. glacialis* was identified by applying morphological methods and sequencing of 18s rDNA, (SSU) and large subunit, 28s rDNA (LSU). Cultivation was performed in a temperature (4 °C) and light (scalar irradiance 50 µmol·m^−2^·s^−1^ PAR) controlled room. Illumination was fluorescent tubes (Osram L 58W/954 Daylight). Photoperiod was L:D; 13:11 and cultivation took place in three replicate (A, B, C) 600 mL Nunc cultivation flasks with f/10 enriched medium (pasteurized seawater and Guillards f/2 water enrichment with silicate added). To ensure that the cultures received the same amount of light, the bottle positions were altered randomly once daily. Prior to the start of the experiment the culture was adapted to the light and temperature regime for one week. At the initiation of the experiment the stock was diluted to concentration of 1.5 g chlorophyll *a* per litre. The culturing experiments were terminated when biomass had increased *ca.* six times. The *P. glacialis* cells were thereafter collected onto four burnt (450 °C, 5 h) GF/C-filters. One filter was taken aside for immediate extraction (same day, first analysis). The remaining filters were immediately flash frozen in liquid nitrogen and kept at −78 °C until the second analysis took place.

Prior to the MS analysis metabolites were extracted with 6 mL 70% aqueous methanol, incubated by shaking in darkness for four hours at 4 °C, and centrifuged at 4000 rpm at 5 °C for 15 min. During the first analysis nine aliquots were analyzed. During the second (after storage at −78 °C for one week) and third (storage at −20 °C for one additional week) five aliquots from each of the three cultivation replicates (A, B, C) were analyzed by HR-MS.

### 3.2. HR-MS Analysis

Mass spectra were acquired using a Waters LCT Premier time-of-flight mass spectrometer equipped with an electrospray ion source. The crude extracts were introduced to the mass spectrometer using a Waters 2795 Alliance HT HPLC without any column. The HPLC pumped a mobile phase consisting of 50% aqueous acetonitrile containing 0.05% formic acid at a flow rate of 50 µL·min^−1^ into the ion source, and aliquots of 50 µL of the extracts were loaded into the flowing mobile phase. The mass spectrometer was operated in the W-mode (both reflectrons active), and at capillary and cone voltages of 2600 and 70 V, respectively. The desolvation chamber was kept at 250 °C and the ion source at 150 °C, while the desolvation gas flow rate was 300 L·h^−1^ and the nebulizer gas flow rate was 5 L·h^−1^. Leucine-enkephalin was infused trough the reference probe and used as lock mass for internal calibrations throughout the data acquisitions. Prior to each analysis day the instrument was tuned to a resolution of at least 10,000 FWHM and calibrated using sodium formate. Data was acquired in the positive ion (ES+) mode, and the mass range was set to 100–1500 *m/z*. One injection was performed for each sample.

### 3.3. Data Processing

A common problem with MS data is that the intensity of an analyte signal (change in instrument response to presence of ions) varies. The reproducibility of a qualitative MS-analysis can be critically dependent on signal intensity [12]. We normalized the *m/z* signal strength (s) between samples according to the formula (1):

(1) $$\text{s }=\;\frac{x\;-\text{ μ}}{\text{σ}}$$

where *x* = sample signal strength, μ = mean of all samples and σ = standard deviation of samples. 

When analyzing MS data for metabolic profiling there are two ways of setting a threshold for signal strength: One is to recalculate *m/z* signal strength as % of the strongest signal (base peak) and discard signals below a certain threshold value. Another is to set an absolute signal strength (ion count) as threshold.

A relatively large part of the less intense *m/z* signals, especially in the low mass region, is noise (fluctuations in instrument background signal). This makes it difficult to use a fixed threshold to exclude low-intensity signals across the entire mass range. We therefore pooled the normalized data and designed a linear algorithm (standardized signal strength *vs.* mass) for the noise threshold. This algorithm was set to accept stronger background noise at higher mass values. The algorithm was tuned by visually inspecting the effect on pooled data from all samples. The final regression line was given by Equation (2):

Standardized signal strength = 0.6316 − (0.0005 × *Mw*) (2)

The original number of centroided *m/z* signals from the MS was between 1896 and 1901, and after the filtering of raw data between 1409 and 1420 *m/z* signals were retained from each sample. In the following processing of data we applied a self-programmed software application (programmed in C+) to compare samples with respect to detected *m/z* signals. The program compared all *m/z* signals in each sample with all *m/z* signals in other samples and arranged this in a matrix where exactly the same *m/z* signal detected in two compared samples was reported as one “hit”. The analysis (comparison) was performed with a mass accuracy of 150 ppm. 

## 4. Conclusions

Interpretation of MS analyses are normally performed by manual inspection or aided by statistical software packages, most often some PCA analysis variant. If large amounts of samples are to be analyzed our conclusion is therefore that, if direct infusion high-resolution mass spectra obtained from biological samples are to be analyzed, great care should be executed when interpreting the results. The same sample should be run as soon as possible after extraction and preferably the number of freeze/thaw cycles should be kept at a minimum. It is also crucial that the same samples are analyzed several times by the MS in order to secure proper statistical analysis of the data. Results must be interpreted with care considering that method and instrument accuracy and compound degradation after freezing can introduce at least 20% variability in some of the detected *m/z* signals. In order to get valid comparisons of metabolic profiles between samples it is paramount that appropriate protocols are implemented [19,20].

## References

[1] Gamache P.H., Meyer D.F., Granger M.C., Acworth I.N. (2004). Metabolomic applications of electrochemistry/mass spectrometry. J. Am. Soc. Mass Spectrom..

[2] Mamas M., Dunn W.B., Neyses L., Goodacre R. (2011). The role of metabolites and metabolomics in clinically applicable biomarkers of disease. Arch. Toxicol..

[3] Chernushevich I.V., Loboda A.V., Thomson B.A. (2001). An introduction to quadrupole-time-of-flight mass spectrometry. J. Mass Spectrom..

[4] Imatani K. (2008). Advances in Accurate-Mass TOF and Q-TOF LC-MS Systems. Am. Lab..

[5] Liu G.-N., Zhu Y.-H., Jiao J.-G. (2009). The metabolomics of carotenoids in engineered cell factory. Appl. Microbiol. Biotechnol..

[6] Smedsgaard J., Nielsen J. (2005). Metabolite profiling of fungi and yeast: From phenotype to metabolome by MS and informatics. J. Exp. Bot..

[7] Jamers A., Blust R., de Coen W. (2009). Omics in algae: Paving the way for a systems biological understanding of algal stress phenomena?. Aquat. Toxicol..

[8] Lei Z., Huhman D.V., Sumner L.W. (2011). Mass Spectrometry Strategies in Metabolomics. J. Biol. Chem..

[9] Lu W., Bennett B.D., Rabinowitz J.D. (2008). Analytical strategies for LC-MS-based targeted metabolomics. J. Chromatogr. B Anal. Technol. Biomed. Life Sci..

[10] Huseby S., Degerlund M., Zingone A., Hansen E. (2012). Metabolic fingerprinting reveals differences between northern and southern strains of the cryptic diatom *Chaetoceros socialis*. Eur. J. Phycol..

[11] Huseby S., Degerlund M., Eriksen G.K., Ingebrigtsen R.A., Eilertsen H.C., Hansen E. (2013). Chemical diversity as a function of temperature in six northern diatom species. Mar. Drugs.

[12] Gika H.G., Theodoridis G.A., Wingate J.E., Wilson I.D. (2007). Within-day reproducibility of an HPLC-MS-based method for metabonomic analysis: Application to human urine. J. Proteome Res..

[13] Allwood J.W., Erban A., de Koning S., Dunn W.B., Luedemann A., Lommen A., Kay L., Loescher R., Kopka J., Goodacre R. (2009). Inter-laboratory reproducibility of fast gas chromatography-electron impact-time of flight mass spectrometry (GC-EI-TOF/MS) based plant metabolomics. Metabolomics.

[14] Rosenling T., Slim C.L., Christin C., Coulier L., Shi S., Stoop M.P., Bosman J., Suits F., Horvatovich P.L., Stockhofe-Zurwieden N. (2009). The Effect of Preanalytical Factors on Stability of the Proteome and Selected Metabolites in Cerebrospinal Fluid (CSF). J. Proteome Res..

[15] Kozikowski B.A., Burt T.M., Tirey D.A., Williams L.E., Kuzmak B.R., Stanton D.T., Morand K.L., Nelson S.L. (2003). The Effect of Freeze/Thaw Cycles on the stability of compounds in DMSO. J. Biomol. Screen..

[16] Chang B.S., Kendrick B.S., Carpenter J.F. (1996). Surface-induced denaturation of proteins during freezing and its inhibition by surfactants. J. Pharm. Sci..

[17] Fogg G.E., Calvario-Martinez O. (1989). Effects of bottle size in determinations of primary productivity by phytoplankton. Hydrobiologia.

[18] Barofsky A., Vidoudez C., Pohnert G. (2009). Metabolic profiling reveals growth stage variability in diatom exudates. Limnol. Oceanogr. Methods.

[19] Sangster T., Major H., Plumb R., Wilson A.J., Wilson I.D. (2006). A pragmatic and readily implemented quality control strategy for HPLC-MS and GC-MS-based metabonomic analysis. Analyst.

[20] Cutillas P.R., Timms J.F. (2010). Approaches and Applications of Quantitative LC-MS for Proteomics and Activitomics. LC-MS/MS in Proteomics. Methods and Applications. Series: Methods in Molecular Biology.

